# How do online learners study? The psychometrics of students’ clicking patterns in online courses

**DOI:** 10.1371/journal.pone.0213863

**Published:** 2019-03-25

**Authors:** Mohini Tellakat, Ryan L. Boyd, James W. Pennebaker

**Affiliations:** Department of Psychology, The University of Texas at Austin, Austin, TX, United States of America; Indiana University Bloomington, UNITED STATES

## Abstract

College students’ study strategies were explored by tracking the ways they navigated the websites of two large (Ns of 1384 and 671) online introductory psychology courses. Students’ study patterns were measured analyzing the ways they clicked outside of the regularly scheduled class on study materials within the online Learning Management System. Three main effects emerged: studying course content materials (as opposed to course logistics materials) outside of class and higher grades are consistently correlated; studying at any time except in the late night/early morning hours was strongly correlated with grades; students with higher Scholastic Aptitude Test (SAT) scores made higher grades but accessed course materials at lower rates that those with lower SATs. Multiple regressions predicting grades using just SATs and click rates accounted for almost 43 and 36 percent of the grade variance for the Fall and Spring classes respectively. Implications for using click patterns to understand and shape student learning are discussed.

## Introduction

Almost 28% of all higher education students take online courses, and these numbers are continuing to rise [1]. Many institutions are transitioning traditional classes to online platforms to reduce costs and/or improve learning [2]. As more classes move to the online world, we are positioned to better track and understand how students think and learn in ways that cannot be achieved in traditional classrooms.

The current research focuses on one of the most basic behavioral patterns that students engage in: mouse-clicks. In an online course, students access class study materials, videos, quizzes, chats, study tips, and old exams via a Learning Management System (LMS; e.g., Blackboard, Canvas). Within an LMS, students must navigate class webpages by clicking web links or URLs. Each mouse click is typically logged and paired with identifying information (e.g., student IDs) and a time stamp. By analyzing clicks, we can quantify the students’ approaches to studying, problem solving, and information search over the entire semester.

### Learning about online learners

The proliferation of online classes has led to a renaissance in education-related research methodologies. In many ways, modern research on students in online courses is analogous to classic research in traditional face-to-face classes. Learner-focused research topics such as demographic influences on course success [3], and instruction methods [4,5] have remained common areas of study, translating nicely from traditional classrooms to various types of online teaching formats.

The evolution of online learning platforms has helped to open the field to experiments with small to very large samples relying on methods such as extensive A/B testing that can simultaneously address both explicitly psychological and infrastructural research questions [6,7]. Although the growth of learning analytics is often attributed to massive open online courses, or MOOCs [8,9], a new generation of online formats are beginning to provide insights into *how* students operate within a system (e.g., through optimal user experience designs), but at some cost to the focus on human elements of learning and behaviors from a psychological/psychometric perspective [10,11]. For example, Reich [12] questioned the distinction between learning and simply clicking within a MOOC interface, in effect suggesting that merely predicting outcomes from high-volume datasets does not help us to understand *how* the outcomes are achieved. Indeed, much of the research in learning analytics can prioritize institutional concerns over student psychology, mirroring the shift in the meaning of the term “learning analytics” over the decades [13].

If we are to make the most of the sheer computational power that can be leveraged against data from students who are learning in online systems, it is imperative to first *understand* the basic psychology of students as captured through their learning behaviors.

### Current research

The current research explores the psychometrics of the most basic online learning behavior–LMS clicks–to provide an enhanced framework for understanding *how* student psychology is reflected in their clicking behaviors. At the outset, it is important to emphasize that we seek to understand studying and performance in a relatively traditional university setting that relies on a novel synchronous online format. Unlike a MOOC, the current research relied on a synchronous massive online course (or SMOC) [14] wherein full time college students would all watch and participate in the course at the same time it was broadcasted live (during the Fall semester) or rerun (the subsequent Spring semester). Most important, the SMOC allowed us to track the ways students searched for course-related material outside of class to help them master the content of the course.

The first step to understanding how college students learn in online environments is to measure their LMS behaviors outside of class, which we call studying. Studying can be defined as devoting time and attention to acquiring knowledge about a subject. As mentioned earlier, the degree to which clicking reflects actual studying has been debated in recent years, with some suggesting that clicking is simply “effort” being put in by students [12]. While clicking must reflect effort to some degree, it can also be conceptualized as a reflection of studying itself. In a system where all materials are online, any engagement with the class outside of classroom hours must be considered some degree of engagement with the learning process. In effect, exhibiting behaviors that capture the intent to gain knowledge are still meaningful in terms of studying as a process. By measuring this real-world behavior, we can examine how different study patterns might affect a student’s ability to achieve academic success. We address four broad questions about clicking behaviors to gain foundational insights into the psychometrics of studying:

#### Research question 1: Is general clicking behavior stable over time?

To what degree do students open online study materials at constant rates over the course of the semester? Test-retest reliability statistics will provide information on the degree to which studying reflects a stable behavioral pattern over time. As stated earlier, we are primarily interested in understanding student learning behaviors from a psychological/psychometric perspective. Temporal reliability of behaviors is a hallmark of differential psychology broadly, and trait psychology more specifically. Given that the “test” itself (i.e., student click behaviors) is objective and does not contain any assessment error, test-retest reliability in this context can be thought of as establishing the degree to which student behaviors on this metric are patterned/regular [15]. Should studying behaviors be found to exhibit high test-retest reliability, then, we will better understand the degree to which studying is a trait-like behavior in psychological terms.

#### Research question 2: Are there meaningful time differences that identify when students are most likely to study?

Most previous studies of student clicking behavior have looked at normative patterns in clicking based on MOOC classes or tracking general behaviors across classes in an LMS system. [16,17,18]. However, individual variations in click patterning are likely to exist between students based on when they access the course or take exams. With the current SMOC format, all students watched the classes and took the exams at the same time which allowed us to begin to identify individual temporal patterns that may differentially correlate with class grades. From an individual differences perspective, variations in when students study may be psychologically meaningful, allowing us to better understand how students differ by viewing their behavior through the lens of their daily schedules. While previous work has also explored temporal patterns of student behaviors within online learning frameworks [19], our current aim is to situate these analyses within an explicitly psychological understanding of variations in human behavior and its relationship to academic outcomes.

#### Research question 3: Do different studying patterns influence course performance?

There is a general consensus that the more people study for classes, the better they perform [20,21]. Additionally, variations in studying techniques appears to be particularly important for success (see the following outstanding summaries [22,23]). However, a primary difficulty exists in objectively quantifying real-world studying behaviors, as self-reports are the most common measurement method. In the current project, we assume that there should be a linear relationship between amount of studying the course material, as measured by the rates of clicking on study materials, and final course grades.

#### Research question 4: Are studying behaviors distinct from aptitude?

It is widely known that college entrance exams such as the Scholastic Aptitude Test (SAT) are general predictor of students’ success in [24,25]. However, *aptitude* is only part of the puzzle, and may chiefly reflect background / past experiences [26,27,28].

What is less clear is the relationship between aptitude and studying behavior. Three possible relationships exist:

1. SAT and studying would be positively correlated, sharing much of the same variance. This prediction assumes that students with higher SAT scores are simply more studious.
2. SAT would be negatively correlated with studying, suggesting that students with higher SATs do not need to study as much or, perhaps, are just simply more efficient studiers.
3. SAT and studying behavior are conceptually distinct constructs reflecting aptitude and achievement striving.

## Methods

All data for the current study were collected from two large and virtually identical Introductory Psychology courses taught using within a SMOC format. The original, or experimental class, was broadcast live in the Fall semester and then was rebroadcast as a replication class to a separate group of students the following Spring. Both classes required students to log into the class via the Canvas LMS to “attend” online lectures. All student activity took place within the LMS, allowing for the virtual tracking every behavior, including URL clicks, student interactions, and so on. Additional details and information on the basic system can be found in Pennebaker et al [29].

Since the data were collected from a standard university course, there was virtually no missing data that could not be explained, and there were clear outcome measures (e.g., course grades), with which MOOC research traditionally has had significant issues. Additionally, the data collected is quite uncommon: two large and completely independent classes separated by a semester that had the exact same course material and class exercises since the second class was a video replay of the first. Because of this, our second sample is a near-perfect replication of the first with a completely different group of students, which allowed for testing the robustness of the findings from the first study.

### Participants

All participants in the current study were fulltime students registered in 1 of 2 large, SMOC courses during either the Fall 2015 (“Experimental Group”; *N* = 1,569) or the Spring 2016 (“Replication Group”; *N* = 736) semesters. Whereas the Fall class was streamed to students live, the Spring class was a “rerun” of the Fall class. As with the Fall course, the Spring class was aired on Tuesday and Thursday afternoons from 3:30–4:45pm. Although the Replication class was a rerun of the Experimental class, the Replication students were aware that the course had been pre-recorded. Nevertheless, they were required to log on to each day’s class at the appointed time so that all students were participating together. All class chats, questionnaires, and online experiments for both classes were conducted identically. Instructors and teaching assistants were available to answer questions as needed.

Students in both semesters took the same number of daily benchmark exams, had the same syllabus, and had to complete the same tasks. Participants were excluded from analyses based on several criteria, including withdrawal from the class and enrollment under a credit/no credit grading system. Additionally, students with no evidence of ever logging into or using the online learning system were dropped from analyses. The final sample sizes were 1,384 and 671 for the Experimental Group and Replication Group, respectively.

#### Demographic composition

For each student, the university’s Registrar provided demographic data such as SAT scores (or their equivalent converted from ACTs), age, sex, and year in college. As can be seen in Table 1, the two classes were quite similar in terms of student composition. While there were some statistically significant demographic differences between the groups, effect sizes were small, which provided the basis for the decision to use the second group’s data as the replication group dataset. Note also that far more students dropped the course in the Fall, 2015 semester (4.97%) than in the Spring, 2016 semester (0.41%). This was likely due to many entering students signing up for the Fall course thinking it was a face-to-face course–which was poorly annotated in the course schedule. This problem was resolved for the Spring semester.

**Table 1:** Sociodemographic information of the participants in the fall and Spring semesters of the 2015–2016 school year.

	Experimental Group	Replication Group	Significance
*N* (original)	1569	736	
% credit/no credit	1.46	1.22	*p* = .64, d = .02
% dropped	4.97	0.41	*p* < .05, d = .23
% no grade	5.35	7.2	*p* = .08, d = .07
*N* (final sample)	1384	671	
Age Mean (SD)	18.8 (1.77)	19.1 (1.56)	*p* < .05, d = .17
Sex			*p* = .68, d = .02
Female	58.7%	60.1%	
Male	40.6%	39.9%	
Did not respond	0.7%	0%	
Race/Ethnicity			
African American/Black	5.5%	4.3%	*p* = .24, d = .05
Asian/Asian American	22.8%	30.6%	*p* < .05, d = .17
Hispanic/Latino	25.8%	28.3%	*p* = .26, d = .05
Anglo/White	40.2%	39.2%	*p* = .56, d = .02
Native American/ Pacific Islander	0.5%	2.1%	*p* < .05, d = .14
Other	5.0%	1.0%	*p* < .05, d = .16
SAT Mean (SD)	1235.3 (151.3)	1255.1 (151.4)	*p* < .05, d = .13
First year students (%)	63.1%	47.2%	*p* < .05, d = .17

*Note*. Descriptive statistics for student demographics represent the final sample for each semester. The “No grade” category is for students who either audited the class or took it pass/fail.

### Procedure

Data for the Fall 2015 Experimental group was collected from August 27, 2015 through December 3, 2015. Data for the Spring 2016 Replication group was collected from January 19, 2016 through May 3, 2016.

#### Ethics

At the beginning of the semester, part of the first class period was devoted to describing all aspects of data collection that would be taking place, including the recording of clicks, saving of chats, class questions, grades, etc. The course instructors disclosed that the data would be used to evaluate teaching and learning and, at the end of the semester, all identifying information would be removed from the data set. Students were required to acknowledge receiving an information form that summarized the data collection and use policy. All students were given the option to have their data removed from the class set at the end of the semester. No students in either class requested that their data be removed.

At the end of the semester, class data were anonymized for all data analyses. A master key linking the anonymized data with student identities is kept by the third author to allow for the collection of additional data as students progressed through the university. The data collection procedures have been approved by the University Institutional Review Board (reference number 2012-07-0064).

#### Class structure

During the first 10 minutes of each class, students completed an 8-question quiz, or “benchmark exam”, where 7 of the questions covered lecture and reading material from the previous class and the 8^th^ was a personalized question for each student based on any missed questions from previous benchmark questions. Following the exams, students tuned into the live or pre-recorded interactive lecture where the professors presented course topics and administered activities. Note that the courses met on Tuesday and Thursday for 1 hour and 15 minutes over approximately 15 weeks. Final grades were based on the highest 22 of the 26 benchmark exams (88% of the final grade) and participation in 4 writing assignments (12% of the final grade). There was no final exam.

All readings and supplemental material were online and consisted of relevant websites such as Wikipedia, open source chapters, newspaper, magazine, or journal articles, as well as videos such as course demonstrations, TED talks, etc. All quizzes were open-book and allowed students to go back to their notes or the course material. Most quiz items were multiple-choice conceptual questions that required students to apply concepts to unique problems or compare theories or perspectives. Multiple versions of each question were created and a sophisticated cheating detection system was employed that caught students collaborating.

### Measures

#### Student clicks (i.e., studying)

Recall that students took tests, checked on grades, downloaded or were directed to class materials, completed writing assignments, watched lectures, and did much more both in-class and outside of class through the LMS. The LMS recorded each student’s movement throughout the class site by logging where and when students clicked, where each click was linked with the student’s ID, a timestamp for the click, and the URL that the student clicked. To simplify the click data, clicks were classified in 3 overarching manners:

1. *Inside versus Outside of Class*: Clicks were classified as “In Class” if they took place within the timeframe of 3:30pm-5pm on the days class was in session. “Out of Class” clicks occurred anytime outside the window. Students who were logged into the class during the “In Class” timeframe but were not participating in the lectures, activities, and quizzes still had their clicks logged as “In Class”. This behavior would be the equivalent of a student going to a live class and not paying attention to the lecture or participating in discussion. For the purpose of analyzing studying behaviors, only “Out of Class” clicks were used.
2. *URL Categories*: Each click had a designated URL that indicated where on the Canvas LMS a student clicked. A script was run to classify each click into different location categories based on keywords in the URLs associated with the pages and tools available on the LMS such as “Grades”, “Course Materials”, “Survey”, and “External Tools”. In total, clicks were classified into 18 categories based on the content of the URLs (S2 Table).
3. *Pre-Benchmark Time Period*: For each class, a pre-benchmark period was identified as the 39 hours leading up to the quiz starting from midnight the day and a half before class up until the start of class. This period was chosen to compare studying behavior for both the Tuesday and Thursday classes with respect to performance on the daily benchmarks since the amount of time between classes differed for each class day. Because all students tended to click at relatively high rates in the 2 hours before each exam, they were excluded from our analyses of study behaviors. Additionally, the hours after class on the class day were not included as there were relatively high rates of clicking on grades as students were able to check progress after class had ended. Note that inclusion of these data did not change the direction or significance of any of the primary findings.

#### Course grade

The course grade was operationalized as each student’s final grade in the course, which was determined by the average scores of his or her benchmark exams and writing assignments together. Students were allowed to drop their lowest-scoring 4 benchmark exams, which were not counted toward their final grades. Student’s numeric scores were then converted into a letter grade based on an A-F scale (Table 2).

**Table 2:** Distribution of letter grades (percentage).

	A	B	C	D	F
Experimental Group	20.6	35.5	27.5	10.8	5.6
Replication Group	27.3	35.8	23.7	9.1	4.2

*Note*. Letter grades were assigned in a standard fashion, with > = 90% being treated as an A, > = 80% being treated as a B, and so on. Grades were not assigned on a curve, and no extra credit was available to students. Experimental: Mean = 82.40, SD = 9.81; Replication: Mean = 83.56, SD = 10.29. *t*(2053) = 2.43, p < .05. The t-statistic was calculated using all students from both groups.

### Final dataset

The final data set includes total number of clicks, average number of clicks per hour, average number of clicks per web location, age, sex, year in college, ethnicity, SAT scores, and Big 5 personality measures [30] for each student.

## Results

### Is general clicking behavior stable over time?

To assess the test-retest reliability of students’ clicking behavior, Cronbach alphas were calculated for the total number of clicks made by each student across the 26 class days during each pre-benchmark period (i.e., the 39 hours leading up to each class). Results showed extremely high test-retest reliability across both groups (Experimental group α = .93; Replication group α = .94; Mean correlation of click rates: Experimental group r = 0.40, Replication group r = .48). The degree to which students studied before any given class period is reflective of their general studying behaviors for the class throughout the entire semester.

### Are there meaningful temporal patterns in students’ studying behavior?

Given that student studying behaviors exhibited high test-retest reliability, we know that studying is a highly patterned behavior, at least in terms of clicking rate (i.e., high-versus-low amounts of studying). However, it is almost certain that substantial between-student variations exist regarding *when* and *where* those study patterns occur. To understand between-student variations along these dimensions, we adopted a data-driven, component-analytic approach.

#### Time of clicks: When students clicked

A Principal Components Analysis (PCA) with varimax rotation was performed on a 1384 (students, experimental group) × 39 (hours preceding each class) matrix, where each cell of the matrix reflects the average number of clicks that a student made during each hour of the pre-benchmark period. The Kaiser-Meyer-Olkin measure of sampling adequacy was .86, and Bartlett’s test of sphericity was significant, χ^2^(780) = 14014.31, *p* < .001. The degrees of freedom are much lower than the sample because the mean number of clicks per student might not have been more than 0 for a given hour. This analysis resulted in 4 major click behavior components, determined primarily using the “elbow” method of scree-plot analysis.

Significant component loadings were determined by using a minimum cutoff of 0.4 (full PCA results and loadings are presented in S3 Table). To calculate the component scores for each student, their number of clicks from each component’s hours were averaged. For example, the “afternoon” component included clicks from the hours 10am through 2pm; each student’s “afternoon” component score therefore reflects their average number of clicks made during each of these hours for each day leading up to the benchmark exam. Mean number of clicks per student for each component are shown in Table 3.

**Table 3:** Time-based click components.

Click Component	Description	Experimental *Mean* (SD)	Replication *Mean* (SD)
Late-Night	Midnight-4am for both days leading up to the exam	0.16 (.182)	0.17 (.232)
Evening	3pm-Midnight the night before the exam	1.03 (.477)	0.93 (.493)
Afternoon	10am-2pm the day before the exam	0.82 (.507)	0.76 (.515)
Morning	7am-9am for both days leading up to the exam	.28 (.279)	0.28 (.304)

*Note*. The component loadings for each of these categories is in the S3 Table. The mean number of clicks for each of these categories is below 1 because on average, most students are not clicking at any given hour of the day, which drives the means down.

Each component from this analysis can be thought of as the degree to which each student tended to perform clicks in certain “buckets” that contained each hour of the day. While most students studied at least a little bit across multiple “buckets”, the majority typically studied during one time over others. For example, few students were *exclusively* late-night studiers, but a subset of students were *primarily* late-night studiers. This does not necessarily mean that a student who studies primarily late at night does not study at other times of the day as well. However, the majority of their time spent studying was from midnight to 4am.

**Fig 1:**
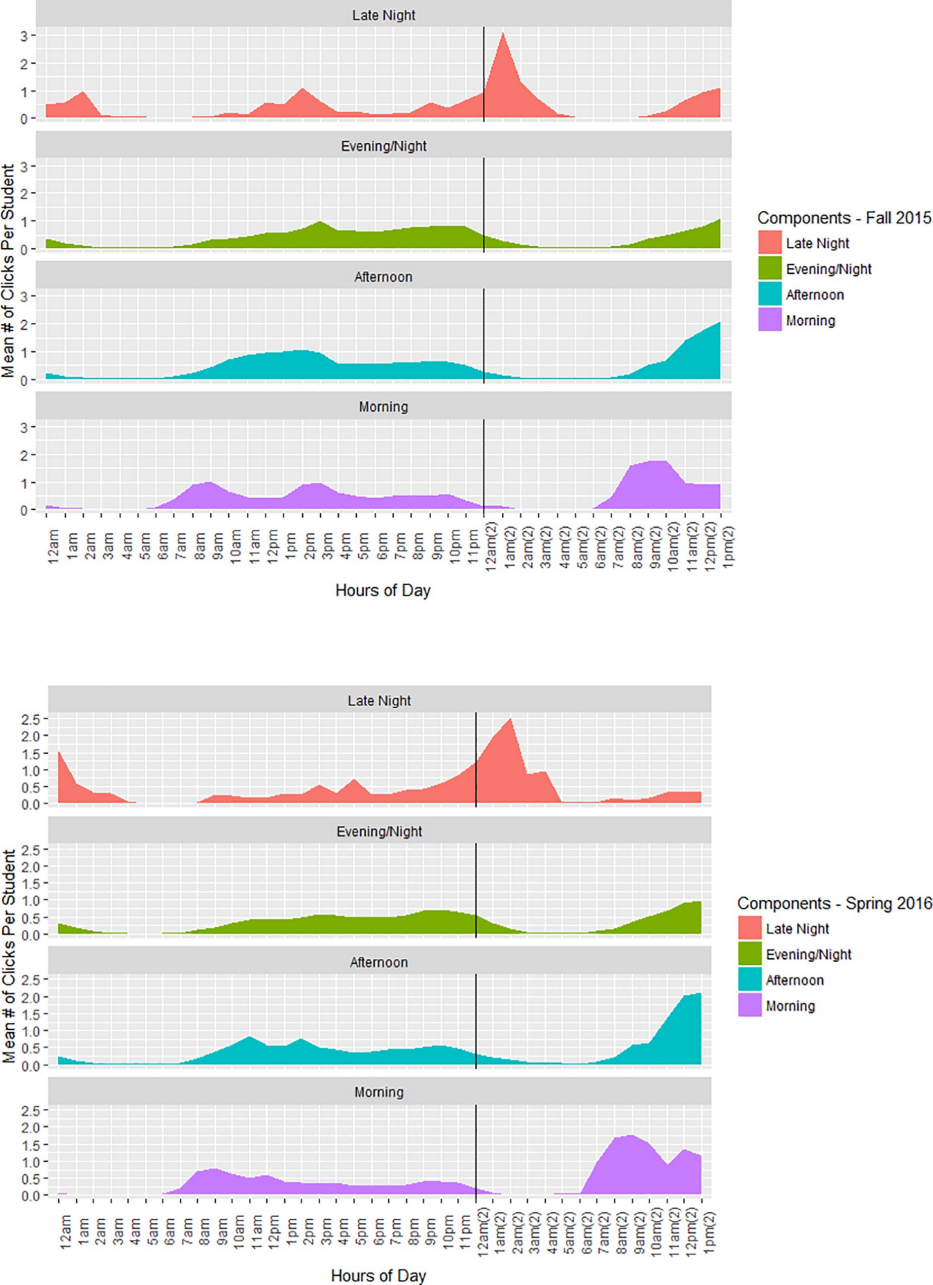
Clicking patterns by time of day during the pre-benchmark period. The vertical line on the graph represents midnight on the day of the benchmark exam and breaks the graph into the 2 days before the benchmark exam. The benchmark exam occurs at 3pm on the second day and is not shown in the graph.

As can be seen in Fig 1, the click components revealed four overlapping study patterns. The late-night studying pattern was associated with elevated studying both the early morning hours the day before the test and especially the day of the test. The evening/night and afternoon patterns both suggest a consistent study regimen at slightly different times of the day, with the afternoon click pattern evincing a last-minute studying sprint. Finally, the morning study pattern is most apparent on the morning of the test. Though the PCA resulted in 4 different click components based on our minimum cutoff criteria, the Afternoon and Evening components had fairly similar and overlapping patterns with small differences in timing, whereas the Morning and Late Night components were distinctly different. Similar patterns were found in both the Experimental and Replication samples.

#### Click location: Where on the webpages students clicked

In addition to *when* students were most active within the LMS, it is important to probe *where* in the system students were active, as well as the relationship between student access to course content and final grades.

A PCA was performed on a 1384 (students, experimental group) × 18 (URL categories) matrix, where each cell of the matrix reflected the number of clicks per student for each link category during the pre-benchmark period. The Kaiser-Meyer-Olkin measure of sampling adequacy was .86, above the commonly recommended value of .6, and Bartlett’s test of sphericity was significant, χ2 (153) = 7596.27, *p* < .001. The degrees of freedom are much lower than the sample because the mean number of clicks per student might not have been more than 0 for a given category. Using the scree-plot method and a varimax rotation, this analysis resulted in 2 major click components: course content clicks and course logistics clicks. Course content clicks were comprised primarily of URLs used to access class materials, such as readings, videos of lectures, and so on. Course logistics clicks, on the other hand, were primarily URLs pertaining to organizational information about the class such as one’s grades, syllabus, and TA announcements. The same components were found in both the experimental and replication groups.

Component loadings were determined by using a cutoff of 0.4 for component inclusion and the component loadings for each of these components are located in the S2 Table. In order to calculate component scores for each student, their total number of clicks belonging to each URL category were averaged into each component. Mean number of clicks per student for each component are shown in Table 4. What this means is that each student’s total number of clicks per component were averaged together for the entire pre-benchmark period.

**Table 4:** Mean number of clicks per person per location component per pre-benchmark period.

Click Component	Experimental Group *Mean* (SD)	Replication Group *Mean* (SD)
Course Content	2.42 (1.10)	1.63 (.739)
Course Logistics	3.28 (1.89)	4.27 (2.58)

*Note*. The component loadings for each of these categories is in the supplemental materials. The mean number of clicks for each of these categories is calculated by taking the average number of clicks across all elements of the component during a single pre-benchmark period, and then was averaged across students. The 2 factors are correlated, *r*(1268) = 0.61, *p* < .05, with each other. Participants were deleted listwise for having missing data. Participants with missing data did not have clicks recorded for the class during the pre-benchmark period.

### Do different studying patterns influence course performance?

#### Clicking and grades

**Fig 2:**
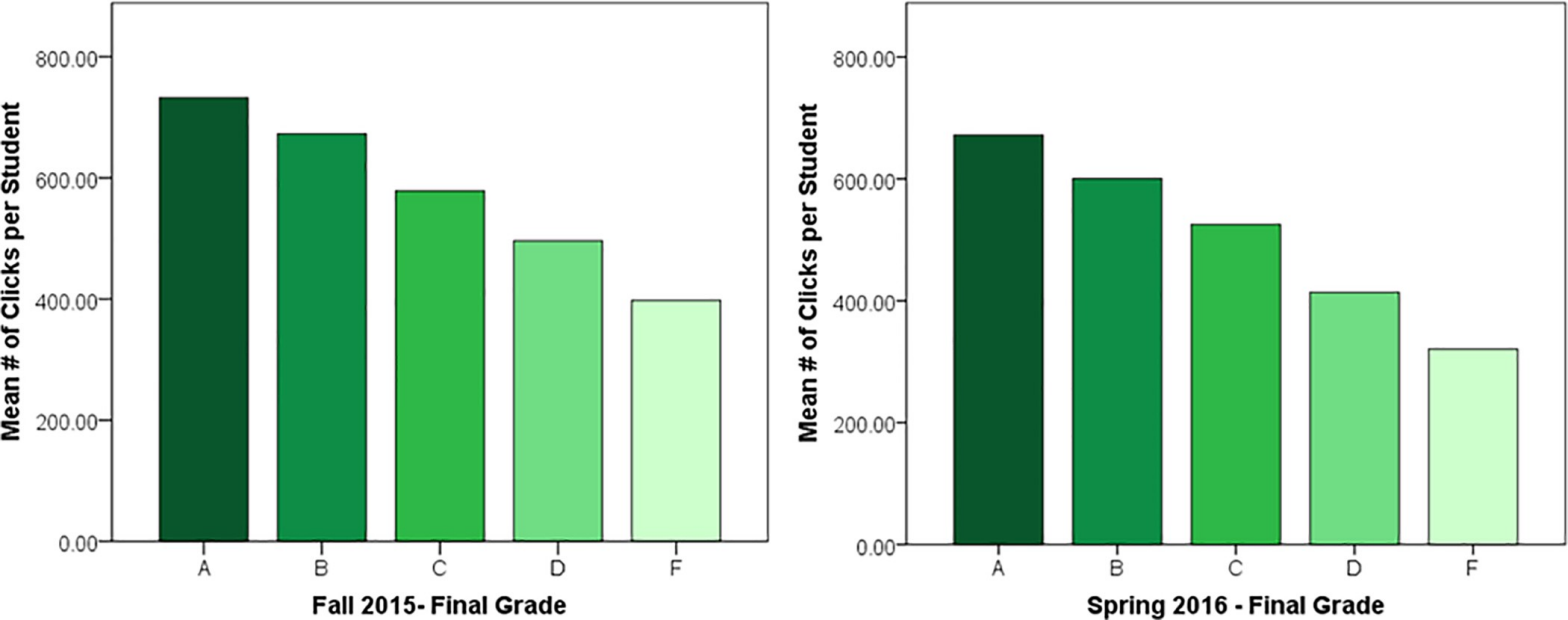
Mean number of clicks per student for every letter grade over the entire semester. (Fall 2015: *F*(4,1265) = 40.159, *p* < .001; Spring 2016: *F*(4,473) = 18.283, *p* < .001). Participants were deleted listwise for having missing data. Participants with missing data did not have clicks recorded for the class during the pre-benchmark period.

To first understand the normative relationship between clicking behavior and grades, a series of Pearson correlations were performed. Results showed significant relationships for both the experimental group, *r*(1268) = .348, *p* < .001, and the replication group, *r*(476) = .367, *p* < .001. In both cohorts, students’ grades were found to increase with a higher number of clicks (see Fig 2). These results show that studying outside of class and higher grades are consistently correlated.

Breaking down the overall clicking behavior into the respective time-based components revealed that studying performed during the evening, afternoon, and morning components positively correlated with grades in the class. Studying that occurred during the late-night period, however, only exhibited weak correlations with grades. See Table 5.

**Table 5:** Correlations between final grades and clicking patterns, by semester.

	Evening	Afternoon	Morning	Late Night
Experimental Group	.312**	.252**	.200**	.067*
Replication Group	.335**	.243**	.138**	.089

Note.

**. *p* < .01.

*. *p* < .05 with *df* = 1268 for Experimental and *df* = 478 for Replication groups. Participants scores were deleted listwise for having missing data. Participants with missing data did not have clicks recorded for the class during the pre-benchmark period.

To better understand the nature of students’ location-based study patterns, the students’ location-based component scores were each correlated with students’ final grades. As shown in Table 6, the *course content* component was more strongly correlated with grade than the *course logistics* component, suggesting that time spent studying the class materials and positive class outcomes are more related to each other than using other parts of the class website and positive class outcomes.

**Table 6:** Location-based clicking components and grade correlation.

	Course Content	Course Logistics
Experimental Group	.409**	.241**
Replication Group	.429**	.250**

Note.

**. *p* < .01 with *df* = 1268 for Experimental and *df* = 478 for Replication group.

Participants scores were deleted listwise for having missing data. Participants with missing data did not have clicks recorded for the class during the pre-benchmark period.

### Are studying behaviors distinct from aptitude?

To build an initial understanding of the relationship between studying behaviors, aptitude, and grades, we performed simple correlations between all 3 measures (Table 7), specifically looking at differences in the location-based click components in these relationships because of the findings seen in Table 6. These simple correlations show significant positive relationships between SAT and grade and clicks and grade, but significantly negative relationships between SAT and clicks.

**Table 7:** Correlations between SAT, location-based click components, and grade.

	SAT and Grade	Clicks and Grade	SAT and Clicks
Experimental Group			
Course Content	.42**	.45**	-.07**
Course Logistics	.42**	.21**	-.06*
Replication Group			
Course Content	.31**	.43**	-.17**
Course Logistics	.31**	.25**	-.09*

Note.

** p < .01

*p < .05

Experimental N = 1249, Replication N = 640.

Participants scores were deleted listwise for having missing data. Participants that did not have SAT scores: Experimental N = 24, Replication N = 31. Other participants with missing data did not have clicks recorded for the class during the pre-benchmark period.

To further understand the unique contributions of studying and aptitude to final classroom performance, a multiple linear regression was conducted wherein grades were predicted from studying as differentiated by location based clicking, aptitude, and the interaction term between these factors (i.e., a moderation model). Each predictor was entered in a separate step in order to examine the relative influence of each variable on student grades. Results are presented in Table 8.

**Table 8:** Regression table for SAT and location components as predictors of grade.

	Model 1: SAT		Model 2: SAT + Content		Model 3: SAT + Content + Logistics		Model 4: SAT + Content + Logistics +SATxContent		Model 5: SAT + Content + Logistics + SATxContent + SATxLogistics	
	B	SE	B	SE	B	SE	B	SE	B	SE
Experimental Sample										
SAT	.028**	.002	.030**	.001	.030**	.001	.043**	.004	.043**	.004
Content Clicks			.018**	.001	.017**	.001	.037**	.006	.042**	.007
Logistics Clicks					0	.001	0	.001	-.006	.004
SATxContent							0	0	0**	0
SATxLogistics									0	0
R^2^	.185		.422		.422		.427		.428	
Replication Sample										
SAT	.021**	.002	.025**	.003	.025**	.003	.018*	.008	.019*	.008
Content Clicks			.026**	.002	.025**	.002	.011	.014	.005	.017
Logistics Clicks					.001	.001	.001	.001	.005	.006
SATxContent							0	0	0	0
SAT x Logistics									0	0
R^2^	.085		.353		.354		.356		.356	

Note.

*p < .05

**p < .01.

Experimental *N* = 1249, Replication *N* = 636. Participants scores were deleted listwise for having missing data. Participants that did not have SAT scores: Experimental *N* = 24, Replication *N* = 31. Other participants with missing data did not have clicks recorded for the class during the pre-benchmark period.

**Fig 3:**
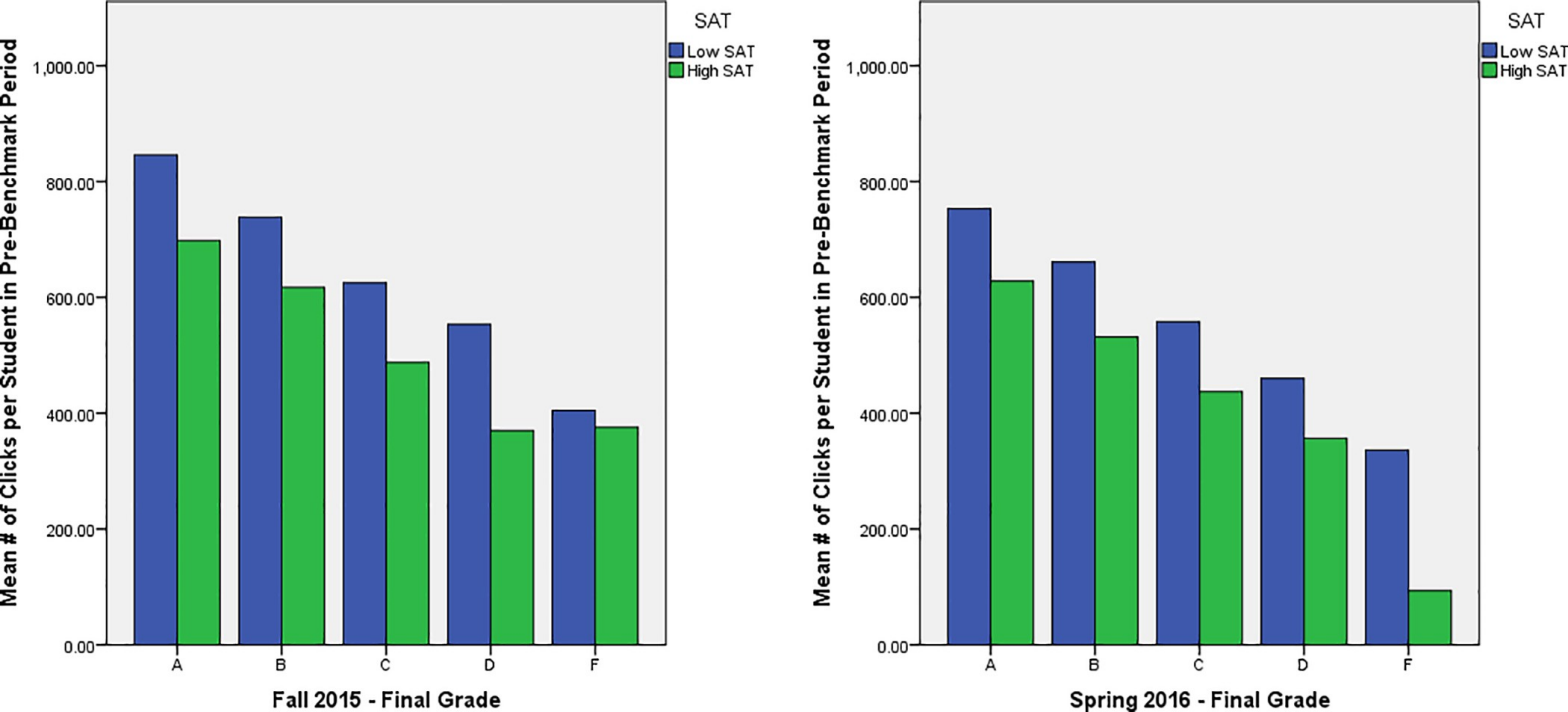
Mean number of clicks per student for every letter grade broken down by high and low SAT scores.

As is apparent in Table 8, course content clicks explain a large part of the variance in grade above and beyond that of SAT scores. The amount of variance in grades that is accounted for by SAT was congruent with the normal range that is often seen in past work (Experimental group: 18.5%; Replication Group: 8.5%). Importantly, course content clicks studying behaviors also accounted for substantial variance in grades (Experimental group: 23.7%; Replication group: 26.8%). Interestingly, though all forms of studying and aptitude exhibited modest negative correlations with each other, there was not a significant interaction in their effect on grades, failing to find evidence that one moderates the linear effect of the other on course performance. For ease of interpreting this relationship, the data were split into High vs. Low SAT scores using a median split and then graphed to compare number of clicks for each grade (Fig 3).

Fig 3 indicates that students who have higher SAT scores are clicking less to attain the same grade as their lower SAT counterparts. This finding speaks to the lack of a significant interaction effect between SAT scores and clicks on grade that we find in our overall model.

## Discussion

The study of student behaviors in online learning environments has mushroomed, yet a rift has grown between traditional psychological research on learning and the area of “learning analytics.” A primary goal of the current study was to begin bridging this gap. By employing traditional psychometric techniques on an objective, high-resolution measure of studying behaviors (i.e., “clicks”), several critical findings emerged that help us to understand not just what learning behaviors might look like, but their psychological implications as well. Perhaps most importantly, this approach also allowed us to see that the role of studying was distinct from aptitude in predicting class performance, with the current research finding no significant moderating effect of one on the other.

As mentioned before, the degree to which clicking reflects actual studying has been debated in recent years. However, the current research shows that the interpretation of the clicking behaviors is conducted in the context of the psychometric and psychological literature, which contributes to the psychometric nature of the analyses. Additionally, the clicking patterns found in these studies are connected to extremely meaningful psychological behaviors (e.g. studying). By relating these patterns to psychologically important traits like aptitude and ability and to important outcomes like academic performance, these findings speak to concepts that are of general interest in the realm of psychology and not only tied to the realm of learning in online courses.

### The psychology of studying behaviors

Overall, the analyses begin to provide unified picture of how students learn in an online environment. Student clicking behaviors exhibited high test-retest reliability and, additionally, showed important differentiation between students both in terms of time of day and click location within the LMS. Critically, results across both years of students revealed a central finding: studying behaviors related to course materials, rather than course logistics, were related to and predictive of classroom performance, above and beyond what SAT predicts. Though all studying and positive academic performance are highly correlated, course content on its own contributes to over 20% of the variance in grade, which suggests that studying course content on its own has an important role in academic performance above and beyond that of studying course logistics and SAT in these online classes.

The fact that timing does not strongly relate to class performance is contrary to past research [31,32]. Though the past findings might reveal students’ perceptions of their study patterns, the results found in the current research are likely to be far more accurate measures of actual study patterns due to the objective nature of the click behavior measure.

Of particular interest is that students with a higher aptitude do not necessarily study more, and that aptitude and studying contribute separately to academic outcomes, which has not always been found in the past [21]. Though both aptitude and studying contribute to academic outcomes, the overall model used to predict grades suggests that the combined influence of SAT and studying is what impacts grades the most.

An important implication is that the more students study, the better they perform. More specifically, students with lower SATs are likely to perform as well as higher SATs by simply working harder. This is not shocking news for educators and, indeed, is certainly consistent with recent research on students’ mindsets about courses [33,34].

### Limitations and future directions

Results from the current study come with several caveats. First, the degree to which the current sample generalizes to others is unknown. Though the SMOC course is taken by a variety of undergraduates at the university (not just students majoring in psychology), the findings may not generalize to all types of classes. Additionally, students across both classes can be considered “high achievers” in general, as the university’s admissions process favors students from the top 7% of their high school class. Relatedly, students were not differentiated by year in college. Future work will likely benefit from broader generalization beyond the current sample. Additionally, the results in the paper are correlational and, as such, causation cannot be established on the basis of these findings alone. Though the current research presents objective measures of study behaviors that were observed in general student behavior in the class, further research is needed to test whether these study patterns actively cause the academic outcomes measured. Nevertheless, the research presented here may be understood as identifying what may be a contributory cause of academic outcomes, as the predictor (i.e., studying) has temporal precedence over the criterion and changes in the former exhibit a relationship with the latter [35].

Aside from addressing such limitations, the potential for research building upon the current work is considerable. The degree to which students’ click behaviors vary across the semester remains an important question, both for identifying students who are struggling to master studying techniques, as well as for identifying potential disengagement from a course (e.g., due to a personal life upheaval), potentially paving the way for improved awareness of students in need.

Relatedly, the development of automated intervention techniques remains a fertile ground to sow. Continued research on all sorts of students’ behaviors, such as diligence and time management skills [36,37], and continued research on interventions targeting students’ studying behaviors [38] will likely allow systems to be created that can train students to optimize their study behaviors, not only through the provision of advice to struggling students, but through training students *how* they can best study (e.g., through algorithm-driven click behavior guidance).

### Conclusion

Studying as measured through out-of-class clicks is a reliable and predictive measure of class outcomes. Students are relatively consistent in the way that they interact with the class materials through their studying. In the future, research on trends in students’ clicking behaviors can help inform us about classroom interactions and specifically find turning points for students, whether that be improvement, or dropping out of a class. The results from this study point to the critical role of learning how to study and study efficiently and are particularly promising for students who enter the university and who may not be as well prepared. Because many more classes are being offered online, understanding the ways in which students interact with these classes and how they are learning will help us create classes that can effectively teach and reach students of all levels.

## Supporting information

S1 Table Correlations between overall clicking and demographic variables. (DOCX)

S2 Table Base rates and factor loadings for click location. (DOCX)

S3 Table Time-based component loadings. (DOCX)

S4 Table Regression table for SAT and overall clicking as predictors of grade. (DOCX)

S5 Table Correlations with overall clicks SAT and grade. (DOCX)
