# Supplementary material for: How do online learners study? The psychometrics of students’ clicking patterns in online courses
Source: PLoS One. 2019 Mar 25;14(3):e0213863. doi: 10.1371/journal.pone.0213863 (PMC6433229; doi:10.1371/journal.pone.0213863)
Supplement: S1 Table — (DOCX) [file pone.0213863.s001.docx]

**S1: Correlations Between Overall Clicking and Demographic Variables**

|  | Experimental Group | Replication Group |
| --- | --- | --- |
| Sex | .10** | .07 |
| Mother’s Education | -.02 | -.04 |
| Father’s Education | .02 | -.05 |
| Computer Literacy | -.12** | -.06 |
| BFI: Extroversion | .02 | .02 |
| BFI: Agreeableness | .05 | .02 |
| BFI: Conscientiousness | .15** | .15** |
| BFI: Neuroticism | .08* | -.02 |
| BFI: Openness | -.17** | -.05 |

*Note.* **p<.01, *p<.05. Sex (1 = Male, 2 =Female). Parents’ Education (1= “Less than 6 years”, 2 = “Less than 12 years”, 3 = “High School”, 4 = “Some College”, 5 = “College Graduate”, 6 = “Master’s Degree”, 7 = “Doctoral Degree”). Computer Literacy (1 = “Not at all”, 2 = “A little”, 3 = “Somewhat”, 4 = “A lot”, 5 = “A great deal”). The correlations suggest that overall clicks per student are positively associated with conscientiousness across both the experimental and replication groups. Computer literacy and Openness are negatively associated with overall number of clicks per student in the experimental group, but the effect doesn’t replicate in the replication group. In the experimental group Females significantly click more than males, but the findings do not replicate in the replication group.
