# Supplementary material for: How do online learners study? The psychometrics of students’ clicking patterns in online courses
Source: PLoS One. 2019 Mar 25;14(3):e0213863. doi: 10.1371/journal.pone.0213863 (PMC6433229; doi:10.1371/journal.pone.0213863)
Supplement: S2 Table — (DOCX) [file pone.0213863.s002.docx]

**S2: Base Rates and Factor Loadings for Click Location**

| Webpage name | Description | Mean # of Clicks per pre-benchmark period (Fall 2015) | Mean # of Clicks per pre-benchmark period (Spring 2016) | Course Content Factor Loadings | Course Logistics Factor Loadings |
| --- | --- | --- | --- | --- | --- |
| Course Materials | Location where all the class materials were located on the course site. | 2.71 | 2.52 | **0.81** | 0.25 |
| Modules | Class readings, handouts, supplemental materials, etc. | 0.57 | 0.89 | **0.44** | 0.12 |
| Pages | Class surveys, questionnaires, and coffee quizzes that students could access at any time. | 2.30 | 1.75 | **0.71** | 0.25 |
| Assignments | Class assignments and benchmark exams | 6.07 | 5.18 | **0.70** | 0.33 |
| Video Archives | Video lectures presented to the class that are accessible after the class period is over | 0.59 | 0.74 | **0.74** | 0.11 |
| Class Launch Page | Links to customized tools for the Psych 301 class – Students go here to watch the lecture live and communicate with class members. Outside of class, clicking here could be a “false click” in that students clicked on this page instinctively since they need to access it every class period. | 1.75 | 1.92 | **0.54** | 0.15 |
|  |  |  |  |  |  |
| Psychology 301 Homepage | Homepage for the Psychology 301 course inside the Canvas LMS | 5.16 | 0.00 | **0.82** | 0.32 |
| Piazza | Discussion board where students were supposed to go to ask questions about class materials to the TAs and instructors for the course. | 0.18 | 0.00 | **0.43** | 0.15 |
| Grades | Displayed grades on benchmark quizzes and other assignments | 3.38 | 2.74 | 0.28 | **0.55** |
| TA Communication | Location where students could go to view classwide announcements about course logistics from TAs | 0.74 | 0.49 | 0.00 | **0.63** |
| Private Student / Instructor Communication | Location where students could go on the LMS to send private messages to instructors of the course. – Students typically used this form of communication to ask grade-related or logistical questions. | 0.98 | 0.91 | 0.00 | **0.73** |
| Downloads | Occurs when students click on a link to download class materials like articles, etc. | 3.10 | 3.75 | 0.28 | **0.52** |
| Canvas Homepage | Homepage for the Canvas LMS for UT Austin | 8.54 | 9.33 | 0.28 | **0.67** |
| Other | All other links | 2.93 | 8.38 | 0.31 | **0.47** |
|  |  |  |  |  |  |
| Syllabus | Course Syllabus – Did not make the cutoff for our factor loadings | 0.00 | 0.00 |  |  |
| Survey | Surveys that students were given either in or outside of class – Did not make the cutoff for our factor loadings | 0.23 | 0.31 |  |  |

*Note.* The webpage name is the location associated with where a student’s click was logged in the system. The pre-benchmark period consisted of the 39 hours leading up to students’ benchmark exams that were completed at the beginning of every class period. The number of clicks per student were logged by location and then averaged across students and across pre-benchmark periods to get the results presented in the table under “Mean # of clicks per pre-benchmark period”.
