# Supplementary material for: How do online learners study? The psychometrics of students’ clicking patterns in online courses
Source: PLoS One. 2019 Mar 25;14(3):e0213863. doi: 10.1371/journal.pone.0213863 (PMC6433229; doi:10.1371/journal.pone.0213863)
Supplement: S3 Table — (DOCX) [file pone.0213863.s003.docx]

**S3: Time-Based Component Loadings**

|  | Component Loadings | | | |
| --- | --- | --- | --- | --- |
|  | Evenings | Late Night | Afternoon | Morning |
| 12am | .360 | **.466** | .007 | -.095 |
| 1am | .200 | **.651** | .013 | -.069 |
| 2am | .083 | **.750** | .039 | .010 |
| 3am | .024 | **.589** | -.020 | .120 |
| 4am | -.067 | **.441** | -.010 | .302 |
| 5am | -.024 | .198 | -.076 | **.561** |
| 6am | .019 | -.013 | .048 | **.621** |
| 7am | .078 | -.099 | .337 | .393 |
| 8am | .027 | -.018 | **.557** | .202 |
| 9am | .133 | -.059 | **.617** | .097 |
| 10am | .173 | -.079 | **.560** | -.018 |
| 11am | .275 | -.011 | **.419** | -.116 |
| 12pm | .367 | .024 | **.484** | -.088 |
| 1pm | .335 | .057 | **.430** | -.122 |
| 2pm | **.451** | -.002 | .287 | -.032 |
| 3pm | **.561** | .052 | .267 | .122 |
| 4pm | **.611** | .023 | .189 | .076 |
| 5pm | **.576** | .078 | .146 | .011 |
| 6pm | **.639** | -.009 | .151 | .088 |
| 7pm | **.627** | -.007 | .093 | .019 |
| 8pm | **.529** | .118 | .056 | -.018 |
| 9pm | **.660** | .104 | .127 | -.009 |
| 10pm | **.657** | .126 | .104 | -.044 |
| 11pm | **.498** | .305 | -.002 | -.078 |
| 12am(2) | .382 | **.486** | -.052 | -.074 |
| 1am(2) | .171 | **.673** | .019 | -.085 |
| 2am(2) | .062 | **.741** | -.009 | -.047 |
| 3am(2) | -.059 | **.699** | -.035 | .144 |
| 4am(2) | -.067 | **.573** | -.064 | .226 |
| 5am(2) | -.008 | .365 | -.061 | **.584** |
| 6am(2) | .103 | .015 | -.019 | **.750** |
| 7am(2) | .075 | -.063 | .291 | **.598** |
| 8am(2) | -.022 | -.021 | **.503** | .246 |
| 9am(2) | .032 | -.031 | **.551** | .114 |
| 10am(2) | .098 | -.001 | **.559** | .016 |
| 11am(2) | .227 | .016 | **.446** | -.047 |
| 12pm(2) | .207 | .051 | **.478** | -.064 |
| 1pm(2) | .266 | .042 | .351 | -.047 |
| 2pm(2) | .277 | -.005 | .170 | .035 |
| 3pm(2) | **.443** | .005 | .148 | .144 |

*Note.* Component loadings over .40 appear in bold. Hours were a part of the component if their component loading was over .40.
