# Supplementary material for: How do online learners study? The psychometrics of students’ clicking patterns in online courses
Source: PLoS One. 2019 Mar 25;14(3):e0213863. doi: 10.1371/journal.pone.0213863 (PMC6433229; doi:10.1371/journal.pone.0213863)
Supplement: S4 Table — (DOCX) [file pone.0213863.s004.docx]

**S4: Regression Table for SAT and Overall Clicking as Predictors of Grade**

|  | Model 1:  SAT | | Model 2:  SAT + Clicks | | Model 3:  SAT + Clicks + SATxClicks | |
| --- | --- | --- | --- | --- | --- | --- |
|  | B | SE | B | SE | B | SE |
| Experimental Sample |  |  |  |  |  |  |
| SAT | .028** | .002 | .031** | .001 | .040** | .004 |
| Clicks |  |  | .015** | .001 | .031** | .007 |
| SAT x Clicks |  |  |  |  | 0 | 0 |
| R^2^ | .185 |  | .352 |  | .356 |  |
| Replication Sample |  |  |  |  |  |  |
| SAT | .021** | .003 | .026** | .003 | .025** | .007 |
| Clicks |  |  | .017** | .002 | .016 | .013 |
| SAT x Clicks |  |  |  |  | 0 | 0 |
| R^2^ | .085 |  | .261 |  | .261 |  |

*Note.* ***p*<.01. Experimental *N* = 1249, Replication *N* = 466. Participants scores were deleted listwise for having missing data.
