# Supplementary material for: How do online learners study? The psychometrics of students’ clicking patterns in online courses
Source: PLoS One. 2019 Mar 25;14(3):e0213863. doi: 10.1371/journal.pone.0213863 (PMC6433229; doi:10.1371/journal.pone.0213863)
Supplement: S5 Table — (DOCX) [file pone.0213863.s005.docx]

**S5: Correlations with Overall clicks SAT and Grade**

|  | SAT and Grade | Clicks and Grade | SAT and Clicks |
| --- | --- | --- | --- |
| Experimental Group | .424** | .348** | -.128** |
| Replication Group | .312** | .367** | -.164** |

*Note.* **p<.01. Experimental N = 1268, Replication N = 466. Participants scores were deleted listwise for having missing data.
